# Xanthan gum intake modifies the colon microbiota profile and causes mild colon inflammation in rats

**DOI:** 10.1371/journal.pone.0347232

**Published:** 2026-04-15

**Authors:** Alessandra B. Silva Rischiteli, Artur Francisco Silva-Neto, Paloma Korehisa Maza, Valter Tadeu Boldarine, Daniel Araki Ribeiro, Carolina Foot Gomes Moura, Marina Gomes Galvani, Raquel Galvão Figuerêdo, Giovana Jamar, Mariana de Moura Dias, Larissa Casemiro Pacheco Monteiro, Tiago Antonio de Oliveira Mendes, Lila Missae Oyama, Daniela Caetano Gonçalves, Claudia Maria Oller do Nascimento

**Affiliations:** 1 Department of Physiology, Universidade Federal de São Paulo, Escola Paulista de Medicina, São Paulo, São Paulo, Brazil; 2 Department of Bioscience, Universidade Federal de São Paulo, Instituto de Saúde e Sociedade, Santos, São Paulo, Brazil; 3 Cancer Metabolism Research Group, Department of Surgery Laboratório de Investigação Médica (LIM26), Faculdade de Medicina, Universidade de São Paulo, São Paulo, São Paulo, Brazil; 4 Laboratory of Clinical Analysis and Genomics, Department of Nutrition and Health, Universidade Federal de Viçosa, Viçosa, Minas Gerais, Brazil; 5 Laboratory of Molecular Biotechnology, Department of Biochemistry and Molecular Biology, Universidade Federal de Viçosa, Viçosa, Minas Gerais, Brazil; Jazan University College of Engineering, SAUDI ARABIA

## Abstract

Xanthan gum is commonly used in the food industry to adjust food consistency and to improve the safety of swallowing liquids and food in people with dysphagia. The pro-inflammatory effect of xanthan gum is acknowledged in the literature. This study aimed to examine the effect of chronic xanthan gum supplementation in the diet on intestinal inflammatory processes in adult Wistar rats at three different doses. After the tenth week of treatment, white adipose tissue (epididymal, retroperitoneal, and mesenteric) was collected, and the distal colon was dissected and processed for cytokine and immunohistochemical analysis. Fecal matter from the colon was used for microbiota analysis. In general, the addition of xanthan gum at all doses promoted an inflammatory state as demonstrated by the high presence of lymphocytes. Also, it modified the content of the pro-inflammatory cytokines IL-1β and TNF-α compared to the control group. Regarding the colon barrier markers, xanthan gum increased the Claudin 2 and ZO-1 levels. The α diversity and relative abundance of *Bacterioidetes* (B), *Firmicutes* (F), F/B ratio were similar among the groups. *Elusimicrobiota* was increased. Our research, using an experimental model, confirmed the clinical assumption that xanthan gum is associated with the development of necrotizing enterocolitis in neonates. We validated the biological mechanism and metabolic pathway in the intestine of the deleterious effect of continuous use of xanthan gum. In conclusion, dietary xanthan gum induced moderate-grade inflammation and modified the colon gut barrier. Recent advances in the study of xanthan gum underscore the need for translational research bridging experimental findings and clinical practice.

## Introduction

Xanthan gum is a polysaccharide used as a food additive with thickener and stabilizer properties. It is commonly used in the food industry to adjust food consistency and also to improve the safety and ease of swallowing liquids and food in people with dysphagia due to both its effective thickness, which endures a wide range of temperature and pH levels, and its tasteless features [1].

In vitro analysis showed that both lipopolysaccharide (LPS) and xanthan gum were efficient in activating lymphocyte cells [2]. Because xanthan gum production involves a fermentation process of the plant pathogen *Xanthamonas campestris*, Inagawa et al. suggested that LPS from these bacteria could be incorporated into xanthan gum during the product purification process [3].

The pró-inflammatory aspect of xanthan gum was also confirmed by Nishimura et al. [4]. They observed a pulmonary inflammatory response after xanthan gum pulmonary aspiration, with increasing neutrophils, in rats. Similarly, Nativ-Zeltzer et al. found pulmonary tissue damage in rabbits exposed to water thickened with xanthan gum [5].

A study showed that treating mice with xanthan gum increased TNF-α production in peritoneal macrophages [6]. Rischiteli Silva et al. treated rats with xanthan gum at doses equivalent to a person with severe oropharyngeal dysphagia for 10 weeks and observed a multisystemic pro-inflammatory effect [7]. These data corroborate other studies in which the use of xanthan gum in the diet altered the expression of pro-inflammatory cytokines [8,9].

Even though side effects and morphofunctional alteration after xanthan gum intake were not observed by most studies [1], some authors associated the necrotizing enterocolitis development with the consumption of xanthan gum‐containing thickening agent infant formula by neonates. The authors suggested that it was related to the immature intestine; xanthan gum could damage the mucosa, activating lymphocytes and macrophages, triggering an inflammatory cascade [8,9].

The community of microorganisms in the gastrointestinal tract, known as gut microbiota, is essential for intestinal function. It is related to ordinary physiological aspects such as immune regulation, nutrient absorption, and maintenance of the epithelial barrier. Dysbiosis, a disbalance concerning the microbial composition, is associated with systemic inflammation [10,11].

In view of reports with neonates, the results of rodents treated with xanthan gum, the prevalence of dysphagia in adults, the need for liquid thickening to keep safe swallowing, and the use of thickeners to cook, it is important to understand the role of xanthan gum supplementation on feeding and intestinal inflammatory processes.

Thus, this research aimed to evaluate the effect of chronic xanthan gum supplementation in the diet at different concentrations on intestinal inflammatory processes in adult Wistar rats.

## Materials and methods

All procedures were approved by the Animal Use Ethics Committee of the Federal University of São Paulo (CEUA no. 3566241018). Thirty-two male Wistar rats from the Central Animal Facility of UNICAMP were kept in the animal care of the Nutrition Physiology Laboratory (UNIFESP) for 21 days, under controlled light cycle conditions (12 hours light and 12 hours dark) and constant temperature (24 ± 1°C), with free access to water and food. At 90 days of age, the 32 rats were distributed into 4 groups, and from 90 to 160 days of age, the animals received the following diets: (1) Control diet (CO); (2) Control diet + 462.5 mg xanthan gum/kg of diet (GX ¼); (3) Control diet + 925 mg xanthan gum/kg of diet (GX ½); (4) Control diet + 1850 mg xanthan gum/kg of diet (GX 1).

Animals received a control diet (Nuvilab, Brazil, 2.79 kcal.g-1) alone or added with xanthan gum according to the groups described above.

FDA’s 2005 body surface area conversion formula [12] was used to calculate the human-to-animal conversion. The xanthan gum dose calculation is described as follows – CO group: no xanthan gum added; GX ¼ group: one-quarter dose: 462.5 mg of xanthan gum/kg of diet to mimic an adult who uses xanthan gum as an ingredient in restrictive gluten-free diets and who also consumes industrialized products that have xanthan gum as a food additive; GX ½ group: half dose: 925 mg of xanthan gum/kg of diet to mimic an adult with mild oropharyngeal dysphagia who uses a xanthan gum-based thickener, but not in all preparations; GX 1 group: total dose: 1850 mg of xanthan gum/kg of diet to mimic an adult with moderate oropharyngeal dysphagia who uses a xanthan gum-based thickener in all liquids and in meal preparation.

Body weight gain and total food consumption were measured over the 10 weeks of treatment. The food efficiency was calculated as the total food consumption (g)/body weight gain (g).

### Experimental procedures

At the end of the tenth week of treatment, the rats were weighed, sedated with ketamine (70 mg/kg) and xylazine (10 mg/kg), and euthanized by cardiac puncture after an eight-hour fast. Immediately, the white adipose tissue (epididymal, retroperitoneal, and mesenteric) and the distal colon were dissected, weighed, placed in liquid nitrogen, and stored at −80°C for subsequent total protein extraction and cytokines and protein analysis. The other portion of the colon was fixed in cork and immediately embedded in paraffin for histological analysis. Blood was collected in dry tubes and centrifuged at 2,000 g for 15 minutes at 4°C, and the subsequent serum was stored at −80°C until biochemical analyses. The adiposity was calculated by the sum of epididymal, retroperitoneal, and mesenteric white adipose weight.

### Serum measurements

Glucose, triacylglycerols, total cholesterol, and HDL-cholesterol serum concentrations were measured using a commercially available enzymatic colorimetric kit (Labtest, Brazil).

### Total protein extraction

The colon was homogenized in 800 μL of chilled extraction buffer (100 mM Trizma Base, pH 7.5; 10 mM EDTA; 100 mM NaF; 10 mM Na_4_P_2_O_7_; 10 mM Na_3_VO_4_; 2 mM PMSF; and 0.1 mg/mL aprotinin). Following homogenization, 80 μL of 10% Triton X-100 was added to each sample. The samples were kept on ice for 30 min and then centrifuged (20,817 g, 40 min, 4˚C), and the supernatant was used for ELISA protocol. Total tissue protein concentration was determined by Bradford (Bio-Rad, Hercules, California), and bovine serum albumin was used as a reference protein. IL1β, IL-6, TNF-α, Claudin 2, Occludin and ZO-1 content in the colon were analyzed by ELISA (DuoSet ELISA, R&D Systems, Minneapolis, MN, USA), following the manufacturer’s instructions.

### Histological analysis of the colon

Sections of the distal colon were collected immediately after euthanasia and fixed in cork and 10% buffered formalin for 48 hours, washed with 70% ethanol for 1 hour, followed by incubation in 70% ethanol overnight.

The samples were processed, embedded in paraffin to block, and subsequently mounted on slides. Slides were prepared with semi-serial sections of 5µm thickness. Hematoxylin and eosin (H&E) staining of the histological sections was used.

Qualitative analysis through morphological description met the following criteria: presence of an inflammatory process, multicellular giant cells, areas of fibrosis, areas of necrosis, metaplasia, dysplasia, and/or adenocarcinoma.

To quantify the degree of inflammation, the tissue was classified by scores, taking into account the intensity of the inflammatory process: 1 – absence of an inflammatory process; 2 – mild inflammation; 3 – moderate inflammation; 4 – severe inflammation [13].

Images were captured using an optical microscope coupled to the imaging system.

**Colon immunohistochemical analysis using ZO-1, Claudin 2, Occludin and TNF-α antibodies:**

ZO-1, Claudin 2, Occludin and TNF-α immunoexpression were evaluated in serial 3-µm sections placed on silanized slides. In the next step, the sections were deparaffinized in xylene, rehydrated in ethanol (99.5%), and pretreated with citric acid buffer (10 mM, pH 6, 0.1 M citric acid, Synth®, São Paulo, Brazil; 0.1 M sodium citrate – Synth®, São Paulo, Brazil) in a microwave for three cycles of five minutes each for antigen retrieval. Then, the sections were incubated overnight with the primary antibodies anti-TNFα, anti-Claudin 2, anti-Occludin, and anti-ZO-1 (ThermoFisher Scientific®, USA), respectively, at the following dilutions: 1:6; 1:700; 1:200; 1:300 (antibody: albumin solution), at 4ºC. The following day, the specimens were subjected to two washes with sodium phosphate buffer (PBS) and the sections were incubated with the secondary antibody for 45 minutes, and then stained with DAB (3,3-diaminobenzine, 0.05%) (DAKO North America Inc®, California, USA) and counterstained with Harris hematoxylin, Sigma®, Missouri, USA).

Once the staining was performed, the proteins were evaluated by the semi-quantitative method from 0 to 3, using the following criteria: 0 – no detectable staining; 1 – < 10% of stained cells; 2 – between 10% and 50% stained cells; 3 – homogeneous staining in >50% of cells according to the methodology of Salim et al. [14].

### Gut microbiota analysis

Following the euthanasia, fecal samples were collected from the colon in a fume hood previously sterilized with UV light for 15 minutes to prevent sample contamination. The feces removed from the colon were placed in sterile microtubes, which were immediately frozen in liquid nitrogen and stored in a −80°C freezer for later DNA analysis. The total DNA of microorganisms present in the fecal samples was extracted using the QIAamp DNA Stool Mini Kit (Qiagen, Hilden, Germany). Subsequently, the DNA was quantified and its quality assessed on a Nanodrop device at 230/260nm.

### 16S rRNA gene amplification and library preparation

The V3–V4 region of the bacterial 16S rRNA gene was amplified using the primers 341F (5′-CCTACGGGNGGCWGCAG-3′) and 785R (5′-GACTACHVGGGTATCTAATCC-3′). Primers were synthesized using pre-adapters compatible with the Illumina platform.

Following the visualization of PCR amplification products on 1.5% agarose gels to confirm successful amplification, the products were purified using AMPure XP Beads (Beckman Coulter Life Sciences).

After purification, the Illumina adapters were added through a second PCR using the Nextera XT Index Kit (Index Primer 1 – N7xx and Index Primer 2 – S5xx). PCR products were purified again using AMPure XP Beads and visualized on 1.5% agarose gels to verify the size and integrity of the libraries. DNA libraries were quantified using a NanoDrop Spectrophotometer (Thermo Scientific) and normalized to equal concentrations. An equimolar pool of all libraries was prepared.

The pooled library was quantified by qPCR using the KAPA Library Quantification Kit for Illumina Platforms (Roche) to determine the final concentration in nM. Sequencing was performed on an Illumina MiSeq platform using paired-end 2 × 250 bp chemistry [15].

### Bioinformatics analysis and microbial diversity and community analysis

The raw sequencing data (files in FASTQ format) were trimmed using the Trimmomatic v 0.36 program [16] with a Phred Quality cutoff of 30. The trimmed data were processed using the DADA2 package version 1.8 [17] on the R platform version 3.6.1 (https://cran.r-project.org/). Data processing followed all the steps recommended by the DADA2 developers, including the following steps: (i) Loading the data into the software; (ii) Trimming the data to remove low-quality bases; (iii) Filtering to eliminate sequences that were smaller than 160 nucleotides; (iv) Removing redundancies and identifying unique sequences; (v) Eliminating chimeras and estimating errors in the sequenced amplicons; (vi) Analysis of the frequency of non-redundant sequences and their taxonomic classification, based on alignments with the Silva release 138 database [18].

The output files from the DADA2 package were used as input for the phyloseq package [19] to analyze the results. Using this package, non-metric multidimensional scaling plots (NMDS) were generated to characterize the variability between replicates and between experimental groups. In these plots, each point represents a sample of the microbiota in the four conditions studied. Using the phyloseq package, the Chao1, Shannon, and Simpson indices were also calculated to compare the biodiversity of the intestinal microbiota. The composition of the bacterial communities was analyzed at the phylum, family, and genus levels.

### Statistical analysis

Statistical analysis was performed in *SPSS Statistic 22.* All data were subjected to normality and homogeneity analysis. For parametric data, the results were expressed as mean ± SEM and were subjected to one-way analysis of variance (ONE-WAY ANOVA) and Tukey’s post hoc test. For nonparametric data, results were expressed as median (minimum value – maximum value) and were subjected to the Kruskal-Wallis test and the Games-Howell post hoc test. Data from histopathological and immunohistochemical analyses were expressed as mean ± SD, as quantification and classification were scores. The level for statistical significance was set at p  <  0.05.

For the microbiota analysis, comparisons between experimental groups were performed using the Kruskal-Wallis and PERMANOVA tests, followed by Dunn’s multiple comparisons. Statistical analyses were performed using the Package Statistical System 20.0 for Windows Evaluation Version (SPSS, 2010) and Scientific Data Analysis and Graphing Software 11.0 (SigmaPlot, 2008), assuming p < 0.05. The composition of the gut microbiota was described in percentage terms. The Spearman correlation was performed to evaluate the relation between gut microbiota composition and the parameters analysed.

## Results

The body weight gain, both food consumption and efficiency, and adiposity were similar among groups (Table 1). Also, the glycemia and serum lipid profile did not differ in any group (Table 2).

**Table 1:** Body weight gain, total food intake, food efficiency and adiposity of rats treated with: control diet (CO), diet added with¼ dose of xanthan gum (GX ¼), diet added with ½ dose of xanthan gum (GX ½) and diet added with full dose of xanthan gum (GX 1). Values are expressed as mean ± standard error of the mean. The number of animals is shown in parentheses.

	CO (8)	GX ¼ (8)	GX ½ (8)	GX 1 (8)
body weight gain (g)	143.07 ± 8.77	154.17 ± 9.36	118.26 ± 5.45	133.04 ± 6.81
food intake (g)	1,892.69 ± 12.2	1,985.86 ± 4.04	1,825.55 ± 7.99	1,826.05 ± 7.67
food efficiency	13.64 ± 0.98	13.18 ± 0.71	15.69 ± 0.79	13.97 ± 0.70
Adiposity (g)	40.95 ± 2.99	42.16 ± 6.33	31.71 ± 3.68	32.66 ± 2.19

The baseline is the CO group – animals that received a control diet

**Table 2:** Glycemia and serum lipid profile of rats treated with: control diet (CO), diet added with ¼ dose of xanthan gum (GX ¼), diet added with ½ dose of xanthan gum (GX ½) and diet added with full dose of xanthan gum (GX 1). For parametric measurements, values are expressed as the mean ± standard error of the mean; for nonparametric measurements, they are expressed as the median (minimum–maximum). The number of animals is shown in parentheses.

	CO (8)	GX ¼ (8)	GX ½ (8)	GX 1 (8)
glycemia (mg/dL)	163.01 ± 10.72	171.61 ± 6.84	171.61 ± 6.84	186.57 ± 8.04
triacylglycerol (mg/dL)	168.56 142.07 - 208.48	162.87 120.29 - 276.23	162.87 120.29 - 276.23	156.68 119.30 - 209.40
cholesterol (mg/dL)	102.23 ± 4.20	111.36 ± 4.71	111.36 ± 4.71	106.90 ± 5.71
HDL-cholesterol (mg/dL)	40.75 ± 2.68	41.37 ± 1.77	41.37 ± 1.77	43.20 ± 6.45

The baseline is the CO group – animals that received a control diet

The IL-1β cytokine content in the colon was higher in GX ½ as compared to the CO and GX 1 groups. No difference was observed in IL-6 and TNF-α content among groups (Table 3). The Occludin protein content was similar among groups. However, the addition of 462.5 mg/kg of diet increased Claudin 2 compared to the other experimental groups and ZO-1 in relation to the control group (Table 3).

**Table 3:** Cytokine and protein content of colon expressed as of rats treated with: control diet (CO), diet added with ¼ dose of xanthan gum (GX ¼), diet added with ½ dose of xanthan gum (GX ½) and diet added with full dose of xanthan gum (GX 1). For parametric measurements, values are expressed as the mean ± standard error of the mean; for nonparametric measurements, they are expressed as the median (minimum–maximum). The number of animals was shown in parentheses.

	CO (8)	GX ¼ (8)	GX ½ (8)	GX 1 (8)
IL-1β (pg/mg of protein)	11.41 ± 0.94	12.53 ± 1.73	18.29 ± 2.68 *	8.87 ± 1.25 ^#^
IL-6 (pg/mg of protein)	4.01 ± 0.63	3.28 ± 0.35	4.69 ± 0.74	3.00 ± 0.44
TNF-α (pg/mg of protein)	0.04 0.02 - 0.05	0.04 0.03 - 0.13	0.04 0.03 - 0.09	0.04 0.02 - 0.06
Claudin 2 (ng/mg of protein)	0.31 ± 0.03	1.04 ± 0.43*	0.28 ± 0.03 ^$^	0.29 ± 0.04 ^$^
ZO1 (pg/mg of protein)	46.04 ± 5.29	83.69 ± 10.69*	50.70 ± 11.83	61.18 ± 10.80
Occludin (pg/mg of protein)	75.16 ± 6.40	79.77 ± 4.43	53.50 ± 6.00	78.21 ± 2.90

* p ≤ 0.05 *versus* CO

# p ≤ 0.05 *versus* GX ½

$ p ≤ 0.05 *versus* GX ¼

### The baseline is the CO group – animals that received a control diet

As described in the Methods section, the degree of inflammation was classified by scores, taking into account the intensity of the inflammatory process: 1 – absence of an inflammatory process; 2 – mild inflammation; 3 – moderate inflammation; 4 – severe inflammation [13].

Table 4 showed that the intestinal inflammation score in the distal colon was statistically significantly increased in the GX ½ and GX 1 groups compared to the control group. The representative images of the immunohistochemical analysis for the TNF-α, Claudin-2, Occludin and ZO-1 are presented in Figs 2–5, respectively.

**Table 4:** The inflammatory score and immunoexpression of TNF-α, Claudin 2, Occludin and ZO1−1 in the colon of rats treated with: control diet (CO), diet added with ¼ dose of xanthan gum (GX ¼), diet added with ½ dose of xanthan gum (GX ½) and diet added with full dose of xanthan gum (GX 1). Values are expressed as mean ± SD. The number of animals is shown in parentheses.

	CO (8)	GX ¼ (8)	GX ½ (8)	GX 1 (8)
inflammatory score	0.2 ± 0.4	0.6 ± 0.9	2.0 ± 1.2 *	1.2 ± 1.3 *
TNF-α	0.4 ± 0.5	2.0 ± 0.7 *	1.4 ± 0.8 *	1.4 ± 0.5 *
Claudin-2	0.8 ± 0.4	2.0 ± 0.7 *	2.0 ± 0.7 *	2.2 ± 0.4 *
Occludin	2.2 ± 0.4	2.0 ± 0.5	2.0 ± 0	2.6 ± 0.5
zona occludens protein 1 (ZO-1)	2.2 ± 0	2.2 ± 0.4	2.2 ± 0.4	2.4 ± 0.5

* p ≤ 0.05 *versus* CO

The inflammatory score was determined by: presence of an inflammatory process, multicellular giant cells, areas of fibrosis, areas of necrosis, metaplasia, dysplasia, and/or adenocarcinoma, taking into account the intensity of the inflammatory process: 1 – absence of an inflammatory process; 2 – mild inflammation; 3 – moderate inflammation; 4 – severe inflammation [13].

**Fig 1:**
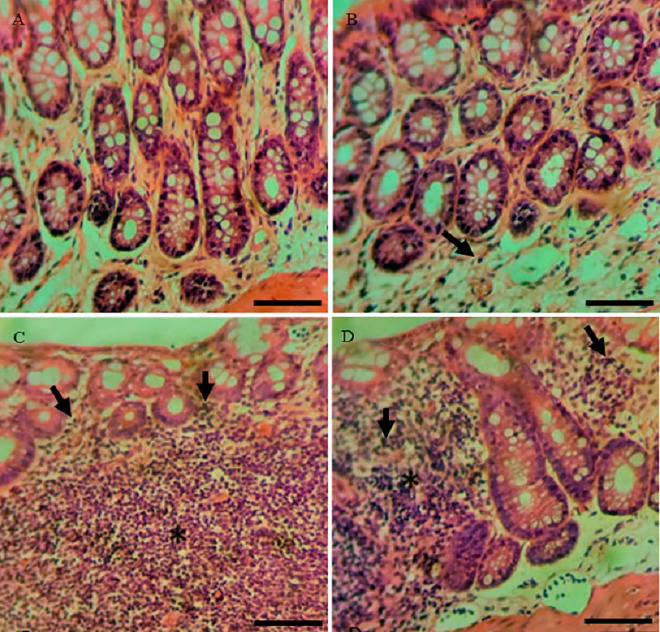
Representative photomicrographs of the distal colon stained with hematoxylin and eosin (H&E) showing histological features of inflammation in the experimental groups: (A) CO; (B) GX ¼; (C) GX ½; (D) GX 1. Inflammatory cell infiltration is indicated by black arrowheads. p ≤ 0.05 versus CO. Scale bars: 50 μm.

**Fig 2:**
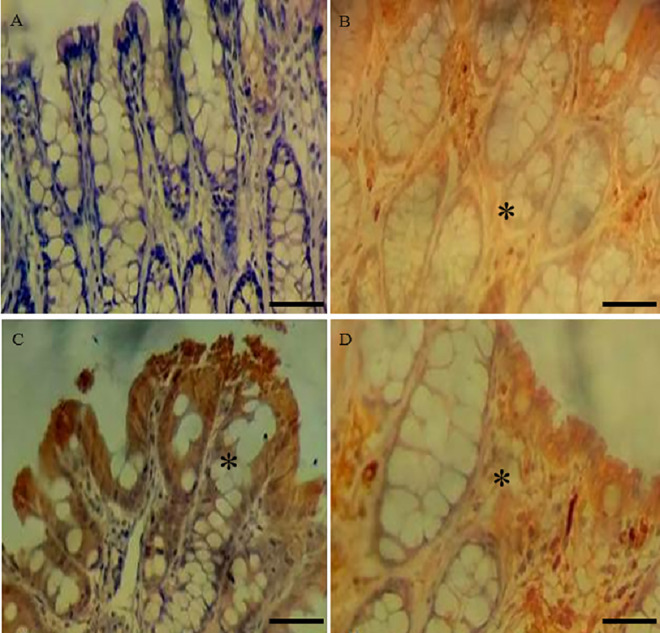
Representative photomicrographs of the distal colon stained by immunohistochemistry with TNF-α antibody. **(A)** CO; **(B)** GX ¼; **(C)** GX ½; **(D)** GX 1. * p ≤ 0.05 *versus* CO. Immunohistochemical staining was performed using the DAB chromogen. Positive staining was identified by brown coloration, indicating TNF-α presence, whereas sections lacking brown staining were considered absent. Hematoxylin was used as a nuclear counterstain.

**Fig 3:**
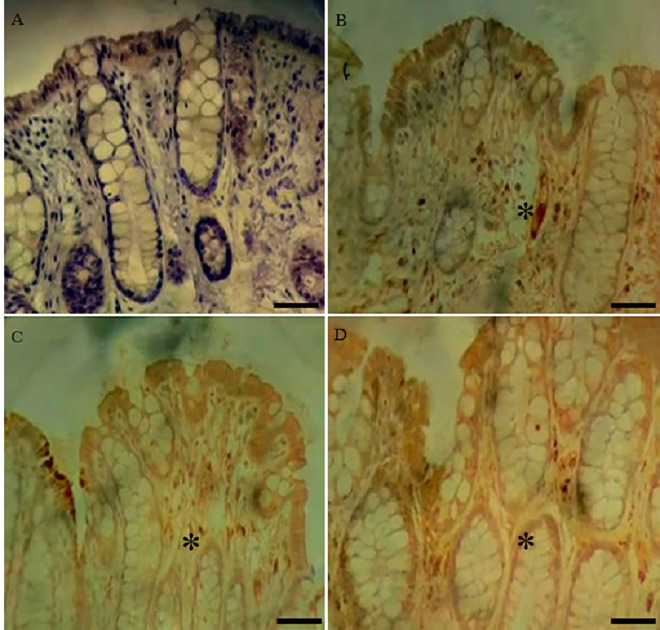
Representative photomicrographs of the distal colon stained by immunohistochemistry with Claudin-2 antibody. **(A)** CO; **(B)** GX ¼; **(C)** GX ½; **(D)** GX 1. * p ≤ 0.05 *versus* CO. Immunohistochemical staining was performed using the DAB chromogen. Positive staining was identified by brown coloration, indicating Claudin-2 presence, whereas sections lacking brown staining were considered absent. Hematoxylin was used as a nuclear counterstain.

**Fig 4:**
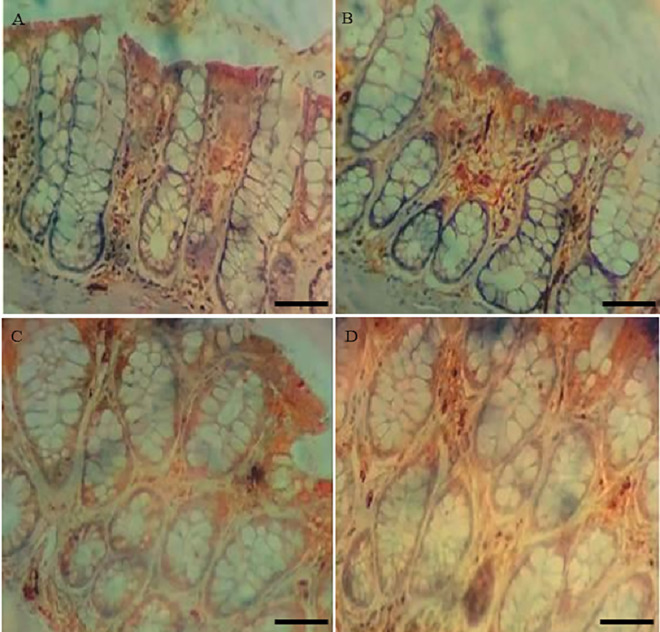
Representative photomicrographs of the distal colon stained by immunohistochemistry with Occludin antibody. **(A)** CO; **(B)** GX ¼; **(C)** GX ½; **(D)** GX 1. Immunohistochemical staining was performed using the DAB chromogen. Positive staining was identified by brown coloration, indicating Occludin presence. Hematoxylin was used as a nuclear counterstain.

**Fig 5:**
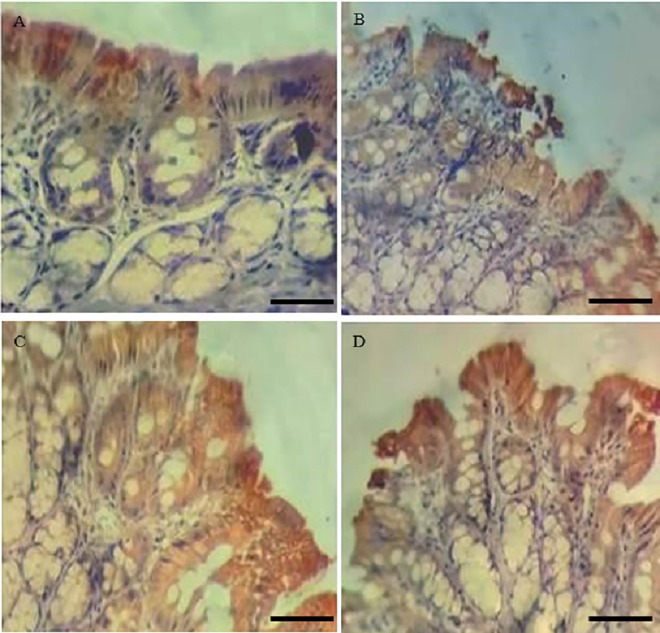
Representative photomicrographs of the distal colon stained by immunohistochemistry with ZO-1 antibody. **(A)** CO; **(B)** GX ¼; **(C)** GX ½; **(D)** GX 1. Immunohistochemical staining was performed using the DAB chromogen. Positive staining was identified by brown coloration, indicating ZO-1 presence. Hematoxylin was used as a nuclear counterstain.

Representative histological images of the treated groups (Fig 1), stained with hematoxylin and eosin, show the presence of inflammatory cells infiltrating the parenchyma of the intestinal wall, particularly in the GX ½ group, followed by the GX 1 dose, when compared to the CO and GX ¼ groups. A magnified lens image shows that the majority of the cells are mononuclear, that is, lymphocytes.

The immunohistochemistry analysis showed a greater presence of TNF-α and Claudin 2 in all groups treated with xanthan gum compared to the control group (Table 4).

**Fig 6:**
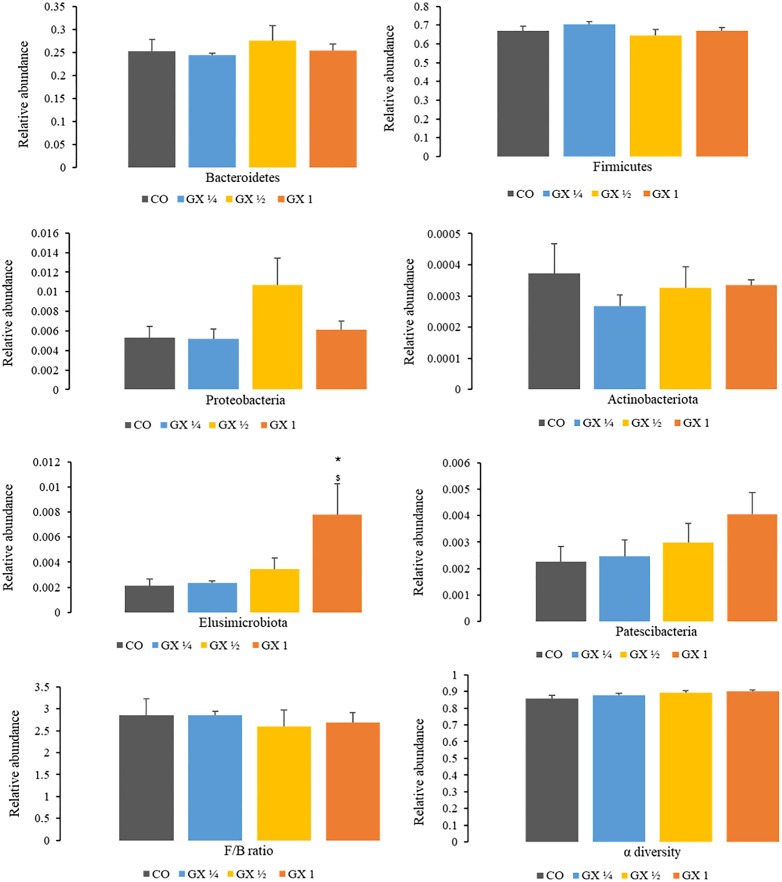
Relative abundance of *Bacterioidetes* (B), *Firmicutes* (F), *Proteobacteria, Actinobacteriota, Elusimicrobiota* and *Pastescibactera* and F/B ratio and alpha diversity of the colon microbiota of rats treated with: control diet (CO), diet added with ¼ dose of xanthan gum (GX ¼), diet added with ½ dose of xanthan gum (GX ½) and diet added with full dose of xanthan gum (GX 1). * p ≤ 0.05 *versus* CO; ^$^ p ≤ 0.05 *versus* GX ¼.

The colonic bacterial microbiota, presented in all experimental groups, belongs to the *Firmicutes* and *Bacteroidetes* phyla. The addition of xanthan gum to the diet did not alter the relative abundance of *Firmicutes* (F) and *Bacteroidetes* (B), nor did it affect the F/B ratio or α diversity (Fig 6).

Regarding the analysis of the less abundant phyla, such as *Proteobacteria*, *Actinobacteria*, *Elusimicrobiota*, and *Patescibacteria*, there was an increase in the relative abundance of the *Elusimicrobiota* phylum (p < 0.04) in the GX 1 group compared to the control and GX ¼ groups. In addition, there was also a slight tendency towards an increase in the relative abundance of the *Patescibacteria* phylum in the GX ½ group compared to the Control group (p < 0.08).

We performed a correlation analysis among the colon microbiota and colon TNF-α, Claudin 2 and Occludin protein content. At the phylum level, a negative correlation was observed between *Cyanobacteria* and TNF-α, and *Proteobacteria* and *Actinobacteria* with Claudin 2. On the other hand, *Elusimicrobiota* was positively associated with Occludin (Table 5).

**Table 5:** Spearman analysis among colon microbiota and colon TNF-α, Claudin 2 and Occludin protein content of rats treated with: control diet (CO), diet added with ¼ dose of xanthan gum (GX ¼), diet added with ½ dose of xanthan gum (GX ½) and diet added with full dose of xanthan gum (GX 1).

	TNF-α	Claudin 2	Occludin
*Cyanobacteria*	r = − 0.503 p = 0.009	ns	ns
*Proteobacteria*	ns	r = − 0.783 p = 0.003	ns
*Elusimicrobiota*	ns	ns	r = 0.581 p = 0.002
*Actinobacteria*	ns	r = − 0.578 p = 0.049	ns

ns – no statistically significant difference

## Discussion

This study brings new findings on the effect of xanthan gum, administered at different doses in the diet of adult rats, on the intestinal inflammatory process and colon microbiota. In an experimental model, it validates the clinical hypothesis that xanthan gum present in infant formulas is associated with the development of necrotizing enterocolitis in neonates, as suggested in 2011 [8,9]. We have shown the biological mechanism and metabolic pathway in the intestine underlying the detrimental effect of prolonged xanthan gum consumption.

Xanthan gum has a low caloric value (0.6 Kcal/g) [20], so it was expected that there would be no statistical difference in body weight gain, feed efficiency and adiposity. Similarly, biochemical parameters showed no differences between the groups. Previously, our group demonstrated that the maximum dose of xanthan gum intake did not modify these parameters compared to the control group [7].

These results indicate that the xanthan gum did not cause any effect on the macronutrient absorption process that occurs in the small intestine, mainly in the duodenum and jejunum [21]. Authors showed that duodenal absorption impairment, as occurred in celiac disease, promoted a decrease in body weight gain and food efficiency [22]. Indeed, major studies regarding the effect of xanthan gum are related to the colon.

When exploring the intestinal histopathological analysis (hematoxylin-eosin staining) of the colon, it is evident that there was a greater infiltration of lymphocytes in the intestinal epithelium. This is important because chronic inflammation is characterized by the presence of mononuclear cells, such as macrophages and lymphocytes [23].

Immunohistochemical analysis of the colon revealed an increase in TNF-α and Claudin 2 levels in all groups that ingested xanthan gum compared to the control group. Increased levels of Claudin 2 are associated with increased intestinal permeability, and elevated TNF-α is associated with epithelial apoptosis and the pathogenesis of inflammatory bowel disease [24].

In an experimental model and under healthy conditions, Claudin 2 is present in the crypts and intestinal villi of developing animals and is restricted to the crypts in adult animals, with a high rate of transport and channel formation [25].

Our immunohistochemical analysis demonstrated the spread of Claudin 2 beyond the crypts and intestinal villi. Claudin 2 elevation has been observed in several intestinal diseases, such as Crohn’s disease, ulcerative colitis, celiac disease, and necrotizing enterocolitis [26–32]. Increased permeability of the intestinal epithelium by increased expression of Claudin 2 in colon crypts is associated with animals and humans that are under the influence of chronic psychological stress [33].

An experimental model using mice and intestinal cells demonstrated that IL-6 increases permeability by modifying TJ, inducing the expression of Claudin-2 [34]. Although we did not observe any effect on IL-6 level, an increase in IL-1β and TNF-α levels was detected. Studies show that TNF-α is a positive regulator in inflammatory bowel diseases by inducing changes in the intestinal barrier that are associated with increased expression of Claudin-2 in intestinal epithelial cultures [35,36], and that increased expression of IL-1β can increase intestinal permeability [37].

Interestingly, the xanthan gum administration promoted an inverted U-shape curve effect on IL-1β concentration in the colon. Although the pattern of IL-1β production in response to injury is dose-dependent, based on the results obtained, we may postulate a hormetic effect of xanthan gum on IL-1β production in colon cells. This is reinforced by the inflammatory process observed in the colon, with greater damage occurring as higher doses are administered [38,39].

A method of encapsulating polyphenols was developed, using a xanthan gum nanocapsule to achieve greater chemical stability and reduced toxicity. The objective was to evaluate transepithelial transport and permeability in the intestine using human colon cells. The xanthan gum nanocapsule resulted in at least a two-fold increase in polyphenol permeability, opening the TJs and promoting the passage of polyphenols, as well as other poorly absorbable bioactive compounds, in an attempt to develop a therapeutic approach [40].

Because xanthan gum increases intestinal permeability, it is not suitable for patients who need continuous use, like those afflicted by dysphagia. In 2011, the FDA (U.S. Food and Drug Administration) issued a warning against the use of SimplyThick® (a xanthan gum-based thickener) in premature infants due to its potential association with necrotizing enterocolitis [9]. Since then, the FDA’s product safety surveillance system, MedWatch program, has been monitoring clinical cases with such an association. Nowadays, clinical concern extends beyond premature infants, including dysphagic patients with serious health conditions, frail elderly individuals, and others.

The gut microbiota can influence health-related outcomes, turning the gut into a vital organ for energy homeostasis. It also plays an important role in maintaining proper gut function and host health. It is in the intestinal epithelium that the mucosal immune system protects the homeostasis of the intestinal microbiota and separates the mucosa from the luminal contents. In the development of inflammatory bowel disease, intestinal dysbiosis and inflammasome activation, elevation of IL-1β and IL-8, and Caspase 1 activity are associated with the autophagy process [41].

Researchers used xanthan gum to microencapsulate probiotics (*Lactobacillus plantarum*) and concluded that the use of xanthan gum negatively modified the intestinal microbiota of the experimental model, reducing *Bifidobacterium* and increasing *Clostridium histolyticum*, which could interfere with treatment with probiotics [42]. In the present study, the xanthan gum ingestion did not change the *Bacterioidetes* (B) and *Firmicutes* (F) relative abundance, including the F/B ratio. Also, it did not modify the α diversity. This supports the idea that xanthan gum did not modify the variety of bacterial species in the colon.

The gastrointestinal tract (GIT) is highly populated by commensal and symbiotic microorganisms, mostly bacteria, but also fungi, archaea, and viruses [43]. The GIT contains ten times more bacteria in its lumen than the number of cells that make up the human body [44].

Dietary components play a key role in determining the characteristics of intestinal colonization. According to Wu et al., the characteristics of the bacteria present in the GIT are influenced by long-term dietary habits and the individual’s phenotype [45].

The relationship between the two dominant phyla, expressed as the *Firmicutes*/*Bacteroidetes* ratio, has been associated with several pathological conditions [46]. Among them, one study has highlighted that the microbiome is related to depression and that there is a bidirectional interaction between the gut microbiota and the brain [47]. Zhang et al. found a positive relationship between serotonin and BDNF (Brain-Derived Neurotrophic Factor) in the hippocampus, with an increase in the phyla *Firmicutes*, *Bacteroidetes*, *Spirochaetes*, *Elusimicrobia*, *Patescibacteria* and *Bacteroidetes* [48].

We performed a statistical correlation analysis among a group of bacteria that make up the gut microbiota and selected systemic and intestinal parameters. As a sign of dysbiosis, a moderate negative correlation was identified between the phylum *Actinobacteria* and Claudin 2 in the colon. In an attempt to activate the systemic protective mechanism to maintain a stable and balanced internal environment, a moderate negative correlation was observed between the phylum *Cyanobacteria* and TNFα in the distal colon, the phylum *Elusimicrobia* and Occludin in the colon, and a strong negative correlation between the phylum Proteobacteria and Claudin 2 in the colon, suggesting an attempt at adaptation/restoration of homeostasis [49–51].

Furthermore, Tomita et al. postulated that the interaction and absorption of the Voglibose (0.2 mg) with xanthan gum reduced the effectiveness of the medication. They emphasize the importance of monitoring the use of xanthan gum by dysphagic patients because it affects pharmacokinetics and decreases the effectiveness of the medication [52].

In conclusion, the addition of xanthan gum to the diet, regardless of the dose, induced a moderate-grade inflammatory process and modified the levels of Claudin 2, an important protein in the formation of tight junctions, in the colon. Additionally, it affected the colonic microbiota, increasing the amount of *Elusimicrobia* at the highest dose ingested, indicating that xanthan gum, even in small doses, was capable of inducing moderate-grade inflammation in the distal colon of rats. However, only high doses were able to modify the colon microbiota. These advances in the study of xanthan gum highlight the urgency of translational research in order to promote a tighter integration between experimental academic research and clinical practice. This may lead to health innovations, including new clinical intervention protocols and disease prevention strategies.
